# Affective Vulnerability to Short Sleep Predicts 10-Year Changes in Chronic Health Conditions

**DOI:** 10.1093/geroni/igaa057.2177

**Published:** 2020-12-16

**Authors:** Nancy Sin, Jonathan Rush, Orfeu Buxton, David Almeida

**Affiliations:** 1 University of British Columbia, Vancouver, British Columbia, Canada; 2 University of Victoria, Victoria, British Columbia, Canada; 3 Pennsylvania State University, University Park, Pennsylvania, United States

## Abstract

We examined daily affective vulnerability to short sleep (i.e., individual differences in the extent that sleeping ≤6h predicts next-day affect) as a risk factor for developing chronic conditions 10 years later. Participants (N=1945, ages 35-85, 57% women) from the National Study of Daily Experiences reported sleep duration and affect in daily diary telephone interviews. Chronic conditions were assessed with a 39-item checklist (e.g., arthritis, hypertension, diabetes). Multilevel structural equation models revealed that individuals with heightened negative affect following short sleep had an increased number of chronic conditions after 10 years (Est.=1.20, SE=.48, p<.01). Positive affective vulnerability (i.e., greater declines in positive affect following shorter sleep vs. longer sleep) was marginally associated with 10-year chronic conditions (Est.=-.72, SE=.40, p=.07). Adding to the well-established connections between sleep duration and well-being across adulthood, these findings suggest that affective vulnerability to short sleep represents a unique risk factor for long-term health as people age.

